# Radiation exposure by medical X-ray applications

**DOI:** 10.3205/000308

**Published:** 2022-03-31

**Authors:** Barbara Buchberger, Katharina Scholl, Laura Krabbe, Ljuba Spiller, Beate Lux

**Affiliations:** 1 Robert Koch Institute, ZIG 2 Evidence-Based Public Health, Berlin, Germany; 2 University of Duisburg-Essen, Institute for Health Care Management and Research, Essen, Germany; 3 University Hospital Bonn, Germany

**Keywords:** diagnostic imaging, radiography, computed tomography, X-rays, radiation exposure, neoplasms

## Abstract

**Background:** Radioactive material and ionising radiation play a central role in medical diagnostics and therapy. The benefit of ionising radiation is opposed by the risk of irreparable damage of the human organism. This risk, especially for developing malign neoplasms, has particularly been investigated in the population surviving the atomic bombing of Hiroshima and Nagasaki, but also increasingly in persons with occupational or medical exposure to ionising radiation.

**Methods:** We conducted a systematic search for publications in English and German in relevant databases in March 2016. Retrievals were screened by two independent reviewers. We included examinations using imaging procedures with ionising radiation. The assessment of methodological quality was done concerning representativeness, risk of bias, and further limitations, and reporting quality was assessed using the RECORD checklist.

**Results:** The systematic searches identified seven cross-sectional, one register, and four cohort studies. An increase in collective effective doses analogue to the increase of computed tomography (CT) examinations could be observed. An increased risk of brain tumours in children after exposition to head CT and by an increase of the number of examinations was shown. For children with predisposing factors, an increased risk of tumours of the central nerve system, leukemia, and lymphoma was found. Furthermore, a general risk for malign neoplasms or haemoblastoma, and a specific risk for lymphoma after CT examinations of different parts of the body could be observed.

**Discussion:** Taking into consideration a mostly unclear representativeness of studies and an unclear or high risk of bias as well as lack of comparability due to different research questions, the validity of results is limited.

**Conclusion:** The risk of bias due to a large number of reference sources must be reduced in studies leading to realistic estimates of collective radiation doses. The risk of CT-induced radiation exposure for children should be investigated by further studies with a follow-up of at least ten years.

## Zusammenfassung

**Hintergrund:** Radioaktive Stoffe und ionisierende Strahlung spielen in der Medizin bei Diagnostik und Therapie eine wichtige Rolle. Dem Nutzen ionisierender Strahlung steht jedoch das Risiko für nicht reparable Schädigungen des menschlichen Organismus entgegen. Dieses Risiko, nach einer Strahlenexposition vor allem bösartige Neubildungen zu entwickeln, wurde ausführlich unter anderem anhand der Population von Überlebenden der Atombombenabwürfe auf Hiroshima und Nagasaki in Japan, aber zunehmend auch anhand beruflich und medizinisch strahlenexponierter Personen untersucht.

**Methoden:** Eine systematische Literaturrecherche nach englisch- und deutschsprachigen Publikationen in relevanten Datenbanken wurde im März 2016 durchgeführt. Die identifizierte Literatur wurde von zwei unabhängigen Gutachtern selektiert. Eingeschlossen wurden vollständige Publikationen über diagnostische Untersuchungen mittels bildgebender Verfahren, bei denen Patienten ionisierender Strahlung ausgesetzt waren. Die Bewertung der methodischen Qualität der eingeschlossenen Studien erfolgte hinsichtlich Repräsentativität, Verzerrungspotenzial und weiteren Limitationen, und die Bewertung der Berichtsqualität anhand der RECORD-Checkliste.

**Ergebnisse:** Durch die systematische Literaturrecherche konnten zwölf Studien identifiziert werden: sieben Querschnittsstudien, eine Registerstudie und vier Kohortenstudien. Hinsichtlich der kollektiven effektiven Strahlendosis zeigte sich über zehn Jahre ein Anstieg analog zur gestiegenen Anzahl von computertomografischen (CT)-Untersuchungen.

Das Risiko für Hirntumoren infolge der Exposition gegenüber Kopf-CT allgemein und durch eine steigende Anzahl von Untersuchungen ist für Kinder erhöht. Im Fall prädisponierender Faktoren zur Entwicklung von Tumorentitäten konnte ein erhöhtes Risiko für Tumoren des Zentralnervensystems, Leukämien und Lymphome festgestellt werden. Ein generelles Risiko für Neoplasien und Hämoblastosen und ein spezielles Risiko für Lymphome nach CT-Untersuchungen verschiedener Körperregionen zeigten sich in einer weiteren Untersuchung.

**Diskussion:** Vor dem Hintergrund der überwiegend unklaren Repräsentativität sowie unklarem bis hohem Verzerrungspotenzial sind die Aussagekraft der Ergebnisse und ihre Vergleichbarkeit durch die Heterogenität der Fragestellungen begrenzt möglich.

**Schlussfolgerung:** Das Verzerrungspotenzial durch unterschiedlichste Referenzquellen muss in entsprechenden Studien gesenkt werden, um kollektive effektive Gesamtdosen realitätsnah schätzen zu können. Das Risiko CT-induzierter Strahlenexposition für Kinder muss in weiteren Studien mit einer Mindestnachbeobachtungszeit von zehn Jahren untersucht werden.

## Introduction

Radioactive material and ionising radiation are playing a central role in medical diagnostics and therapy. Ionising radiation is of very high energy and may cause damage to biological tissue at the atomic and molecular levels. Sources of ionising radiation are natural, but may also be produced by technical means. The application of ionising radiation can be categorised into procedures for diagnostic radiology, nuclear medical diagnostics, and radiographic procedures for therapy. In Germany, the mean effective dose per inhabitant was 1.7 mSv (2011) caused by radiographic examinations, representing a significant proportion of the total collective effective dose of 4.0 mSv [1].

In contrast to radiographic examinations, which are associated with a low level of radiation exposure (<0.01–0.7 mSv), angiographic or computed tomographic (CT) examinations are considerably associated with higher exposure (up to 16.4 mSv) [1].

The Federal Office for Radiation Protection has been collecting the radiation exposure of the population stratified by sources of radiation since 1996. During the period from 1996 to 2012, an increase of exposure by radiographic examinations by 13% was found. Therefore, the increase of CT examinations by 130% during the same period contributed substantially [1].

The benefit of ionising radiation is opposed by the risk of irreparable damage of the human organism. This risk for developing malign neoplasms or haemoblastoma was particularly investigated in the population surviving the atomic bombing of Hiroshima and Nagasaki in Japan. Depending on the distance to the point of bombing, the radiation dose exposing the population could be defined sufficiently. Based on these results, the incidence of malign neoplasms and haemoblastoma was investigated in epidemiologic studies [2], [3], [4].

Recommendations for responsible use of ionising radiation for diagnostic purposes are therefore necessary.

## Research questions

1. How has the radiation exposure of patients in Germany changed in the last 20 years due to technical developments in diagnostic procedures?
2. Are there any alternative procedures with clearly lower radiation exposure compared to standard procedures by the example of a specific indication, and if yes, how often are these procedures alternatively used in Germany?
3. What is the risk of diagnostic procedures using ionising radiation for causing malign neoplasms and haemoblastoma in exposed children in their life course?

## Methods

We conducted a systematic literature search for publications in English and German in the databases of the German Institute of Medical Documentation and Information (DIMDI) (MEDLINE, Cochrane Central Register of Controlled Trails, Cochrane Database of Systematic Reviews, DAHTA Database, Database of Abstracts of Reviews of Effects, Health Technology Assessment Database, NHS Economic Evaluation Database, EMBASE, BIOSIS Previews, EMBASE Alert, SciSearch) via user interface ClassicSearch, and EBSCO (CINAHL Complete, Health Business Elite, SocINDEX) via user interface EBSCOhost in March 2016.

The literature identified was screened by two independent reviewers according to defined inclusion and exclusion criteria. We included full text publications about diagnostic examinations using imaging procedures with ionising radiation exposure to patients. Data extraction into evidence tables was checked by a second reviewer. The same applies for the assessments of methodological quality concerning representativeness, risk of bias, and further limitations as well as for reporting quality which was assessed using the RECORD checklist. Verification and assessments followed international standards of evidence-based medicine.

## Results

The systematic literature searches identified twelve studies: seven cross-sectional studies [5], [6], [7], [8], [9], [10], [11], one register study [12], and four cohort studies [13], [14], [15], [16]. Seven studies investigated national data [5], [7], [8], [9], [10], [11], [12] and one study European data of 36 countries [6] on collective effective doses of ionising radiation by different diagnostic procedures and total collective effective doses. The cohort studies [13], [14], [15], [16] investigated the risk of children having been exposed to ionizing radiation by CT examinations for the development of malign neoplasms or haemoblastoma. The systematic searches identified no studies about radiation exposure in Germany, and the study about 36 European countries [6] did not stratify the results for single countries. Therefore, we could not answer the research questions 1 and 2. Four out of eight studies about collective effective doses were assessed as being representative [7], [9], [10], [12], the representativeness of the other four studies was unclear [5], [6], [8], [11]. Regarding other causes of bias, the judgment was “high potential of bias” for two studies [9], [12], and “unclear risk of bias” for six studies [5], [6], [7], [8], [10], [11]. The assessment of the cohort studies investigating the risk of tumor entities after radiation exposure in children resulted in one study being representative [14], one being not representative [16], and two studies with unclear representativeness [13], [15]. Risk of bias was high in two studies [14], [16] and unclear in the other two studies [13], [15]. Reporting quality was heterogeneous but mostly acceptable.

The investigations about the collective effective doses of single procedures and the total effective doses reported an increase in collective effective doses analogue to the increase of CT examinations for a period of approximately ten years.

An increased risk for the development of brain tumours in children after exposition to head CT in general and by an increase of the number of examinations was shown [13]. For children with predisposing factors for tumour entities, an increased risk for the development of tumours of the central nerve system, leukemia, and lymphoma was found [14]. Another investigation resulted in a general risk for malign neoplasms or haemoblastoma, and a specific risk for lymphoma after CT examinations of different parts of the body [15].

## Discussion

Against the background of a mostly unclear representativeness of studies and an unclear or high risk of bias, the interpretation of the results is difficult.

Regarding the studies evaluating collective effective radiation doses, it should be discussed whether the results are comparable due to the differences in number and kind of radiographic procedures included and due to the differences in study design (cross-sectional and longitudinal studies). In addition, medical databases as well as evaluation systems of health insurances or hospitals were used, differing not only between studies but also within studies, and therefore increasing error and bias potential. Apart from national differences, the comparability of studies is further limited by technical conditions of single devices.

## Conclusions/Recommendations

Concerning the investigation of changes in radiation exposure, the potential of bias due to differing reference sources must be decreased to result in realistic estimates for total collective effective doses. To further examine the risk of CT-induced radiation exposure for children, additional studies with a follow-up of at least ten years are necessary, observing children up to the age of 15. A documentation of each CT examination and its individual dosage, the body part examined, and eventually applicated contrast media should be done and published. A verification of indication should also be reported, stratified for the medical discipline of the indicating physician (pediatrician, radiologist, pediatric radiologist).

## Notes

### HTA report

This article is the short version of the HTA report of the same title [17].

### Competing interests

The authors declare that they have no competing interests.
